# Simplifying PA Quantification: A Unified Enrichment and Reduction Workflow for Retronecine-Based Quantification of Pyrrolizidine Alkaloids in Phyto Samples

**DOI:** 10.3390/molecules31203590

**Published:** 2026-10-09

**Authors:** Benedikt Schwarz, Thomas Jakschitz, Christian W. Huck, Moritz Rubner, Michael Popp, Günther K. Bonn

**Affiliations:** 1 ADSI—Austrian Drug Screening Institute GmbH, University of Innsbruck, Mitterweg 24, 6020 Innsbruck, Austria; 2 Institute of Analytical Chemistry and Radiochemistry, CCB—Center for Chemistry and Biomedicine, Leopold-Franzens University, Innrain 80/82, 6020 Innsbruck, Austria; christian.w.huck@uibk.ac.at; 3 Bionorica SE, Kerschensteinerstraße 11–15, 92318 Neumarkt, Germany

**Keywords:** pyrrolizidine alkaloids, retronecine, nano zirconium silicate, liquid chromatography–mass spectrometry, phyto samples

## Abstract

Pyrrolizidine alkaloids (PAs) are a chemically diverse group of plant-derived toxins whose quantification in botanical materials remains analytically challenging. This study integrates previously established nano zirconium silicate (NZS) enrichment with one-pot lithium aluminium hydride reduction/hydrolysis and LC–HRMS determination of the resulting necine-base response. The workflow targets retronecine- and heliotridine-type PAs and their N-oxides, with results expressed as retronecine equivalents. In *Radix Primulae*, three independent samples were spiked at each of 200, 250 and 300 µg/kg retronecine equivalents and processed through the complete analytical workflow. Mean recoveries were 83.0%, 94.2% and 92.5%, with between-preparation RSDs of 3.12%, 1.55% and 1.96%, respectively. Matrix-matched regression was evaluated using six calibration levels (10, 100, 200, 300, 400, 500; Day-1 R^2^ = 0.99936). Calculated Day-1 LOD and LOQ estimates were 14.94 and 45.27 µg/L. Remeasurement of the same extracts after 24 h, using a separate daily calibration, gave paired concentration changes of −0.67% to +1.23%. Application to a naturally contaminated comfrey sample demonstrated the workflow’s applicability beyond spiked samples, with agreement between the conversion-based result and the summed intact-PA reference approach.

## 1. Introduction

Pyrrolizidine alkaloids are widely distributed in plants, especially in certain families such as Boraginaceae, Asteraceae and Fabaceae [1].

More than 500 individual PA and PA-N-oxide derivatives have been identified across over 6000 plant species [2]. These compounds are primarily secondary metabolites that serve plant defence roles [1]. Within this group, 1,2-unsaturated PAs, typically based on necine types such as retronecine and heliotridine, are of particular toxicological relevance because the C1–C2 double bond is a key structural feature associated with bioactivity and toxicity [3].

The toxicity of 1,2-unsaturated PAs is closely linked to their metabolic activation in the liver. After absorption, these compounds undergo bioactivation mainly by cytochrome P450 monooxygenases to form highly reactive dehydropyrrolizidine (pyrrolic) metabolites that can bind with DNA, resulting in DNA crosslinking as well as DNA protein crosslinking. This bioactivation pathway is central to both their acute hepatotoxic and chronic genotoxic and carcinogenic properties and has been extensively reviewed in mechanistic toxicology studies [4,5,6,7]. Clinically, high-dose or subacute exposures in humans and animals can result in hepatic veno-occlusive disease, characterised by hepatomegaly, ascites and hepatic failure. Numerous outbreaks have been linked to PA-contaminated grains, herbal remedies and teas. Chronic low-dose exposure is associated with the formation of PA-derived DNA adducts and the development of liver tumours in experimental animals, supporting a genotoxic mode of action [8,9,10].

In food and phytomedicine, PAs are detected in a variety of matrices. Large monitoring and occurrence studies, as well as systematic reviews, have reported frequent detection in herbal teas and infusions, honey, some cereal-based products and food supplements containing botanicals from PA producing or contaminated sources [11,12,13,14].

Concentrations span several orders of magnitude, from low ng/kg to mg/kg levels, depending on the botanical source, agricultural and harvesting conditions and processing steps. Contamination of non-producing crops via co-harvested weeds, pollen, or plant debris further increases the number of matrices in which PAs can occur and complicates efforts to attribute exposure to specific botanical sources [3,12,15,16]. Moreover, carryover from livestock consuming PA-contaminated feed has been shown to result in detectable PA levels in animal-derived products such as milk, adding a route of human exposure [17,18].

Recent reviews have highlighted that, despite the growing body of occurrence data, there remain substantial gaps regarding minor PA congeners, seasonal and geographic variability. This underscores the need for analytical strategies capable of simultaneously capturing a broad range of congeners and enabling reliable quantification across diverse concentration ranges and matrices [19,20].

The European Union has established maximum levels for the sum of specified PAs and PA N-oxides in several food categories under Commission Regulation (EU) 2023/915. These limits are category-specific and use lower-bound concentrations, which should not be interpreted as a single threshold applicable to all botanical products [19,21,22].

The European Directorate for the Quality of Medicines and Healthcare (EDQM) introduced a dedicated chapter on pyrrolizidine alkaloids in Supplement 10.6 of the European Pharmacopoeia, which entered into force on 1 July 2021. This chapter defines the analytical target pyrrolizidine alkaloids and allows the use of any suitable chromatographic procedure coupled with tandem mass spectrometry (MS/MS) or high-resolution mass spectrometry. The introduction of this chapter reflects the need expressed by European regulatory authorities after reports from multiple European Pharmacopoeia member states indicated the presence of pyrrolizidine alkaloid-containing plant material as contaminants in herbal medicinal products and food products [23].

Necine-base sum-parameter methods can complement targeted determination of individual PAs by reducing the number of reference compounds needed for routine quantification. However, a result expressed as retronecine equivalents is not numerically equivalent to the regulatory mass sum of intact PAs and their N-oxides. Conversion removes information on individual PA identity and molecular mass and the analytical scope may differ from the regulated analyte list. Accordingly, the present workflow is intended as a complementary screening and sum-quantification approach, not as a replacement for targeted analysis used to assess regulatory compliance.

**Figure 1:**
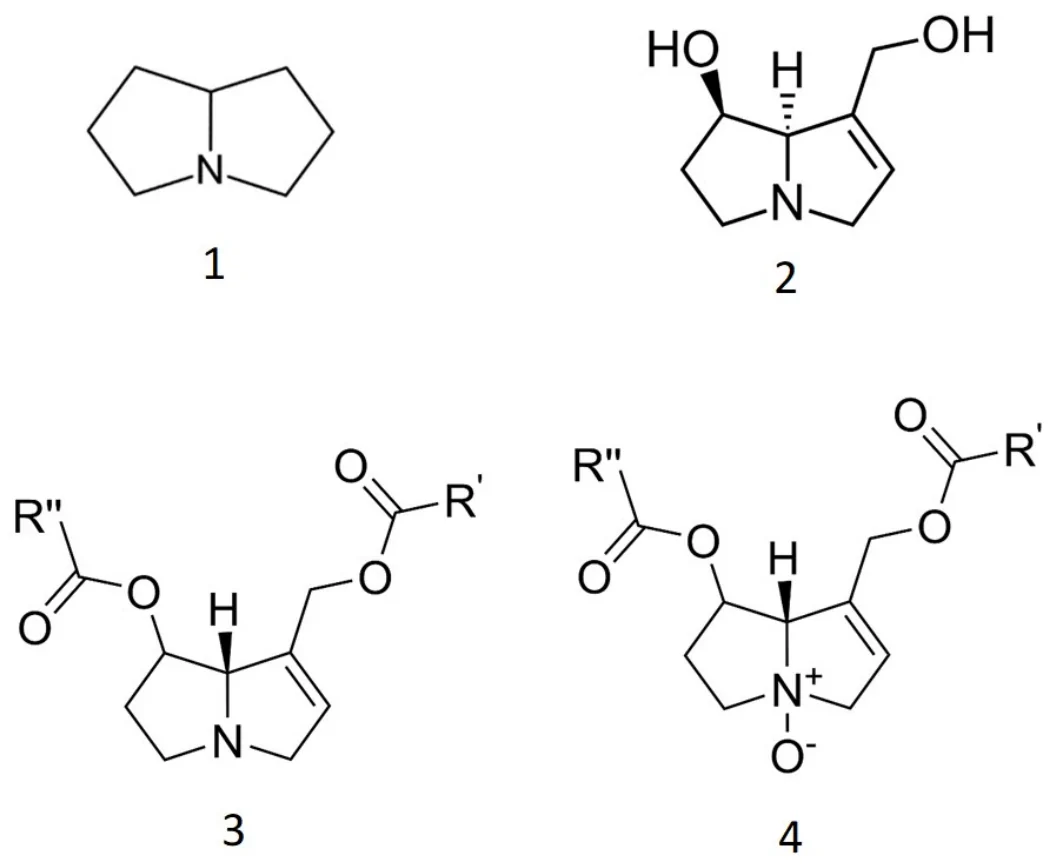
Chemical structures of pyrrolizidine (**1**), retronecine (**2**), a representative PA (**3**) and a representative PANO (**4**).

Established sum-parameter approaches convert esterified PAs to necine-base markers for quantification [24,25,26]. Retronecine (C_8_H_13_NO_2_) is a 1,2-unsaturated necine base containing hydroxyl groups at C7 and C9; pyrrolizidine provides the underlying bicyclic skeleton (Figure 1) [27]. Retronecine-type PAs yield retronecine, whereas heliotridine-type PAs, including heliotrine and heliotrine N-oxide, yield heliotridine. The resulting necine-base response is expressed here as retronecine equivalents. This reporting convention does not imply that all PAs form retronecine or that the individual necine bases are distinguished by precursor mass alone.

Selective enrichment before conversion is intended to reduce the matrix burden entering the reaction and LC–MS analysis. The NZS extraction procedure was adopted from our previously published method for enrichment of intact PAs and PANOs [14]. In the present work, this established cleanup step is coupled with a reduction/hydrolysis concept based on the publication by Cramer et al. [26]. The combined procedure is evaluated directly in *Radix Primulae*, with an additional application to comfrey.

The aim of this study was to evaluate the analytical performance of the integrated workflow for a representative mixture of retronecine- and heliotridine-type PAs/PANOs in a botanical matrix and to examine its potential as a complementary sum-parameter method.

## 2. Results and Discussion

The unified enrichment and reduction/hydrolysis workflow was evaluated in *Radix Primulae* using recovery, calibration, sensitivity and precision experiments. Throughout this manuscript, sum-parameter results are expressed as retronecine equivalents of the measured necine-base response. They refer to the investigated retronecine- and heliotridine-type PAs/PANOs and do not represent an experimentally established total for every naturally occurring PA.

### 2.1. Recovery, Precision and 24 h Remeasurement

To assess recovery of the complete analytical process, *Radix Primulae* was spiked before extraction with a defined PA/PANO mixture at 200, 250 and 300 µg/kg retronecine equivalents. Three samples were prepared independently at each concentration, giving nine preparations in total. Each final extract was injected twice and the mean peak area of the two injections was used to calculate one result per preparation. The same nine extracts were measured again in duplicate after 24 h and quantified using the calibration measured on that day.

On Day 1, mean recoveries at 200, 250 and 300 µg/kg were 83.0%, 94.2% and 92.5%, respectively (Table 1). The corresponding ranges across the three preparations were 80.6–85.8%, 93.3–95.9% and 90.4–93.7%. Between-preparation RSDs, calculated using the sample standard deviation (*n* − 1), were 3.12%, 1.55% and 1.96%. These RSDs describe the dispersion of independently prepared samples after averaging duplicate injections.

**Figure 2:**
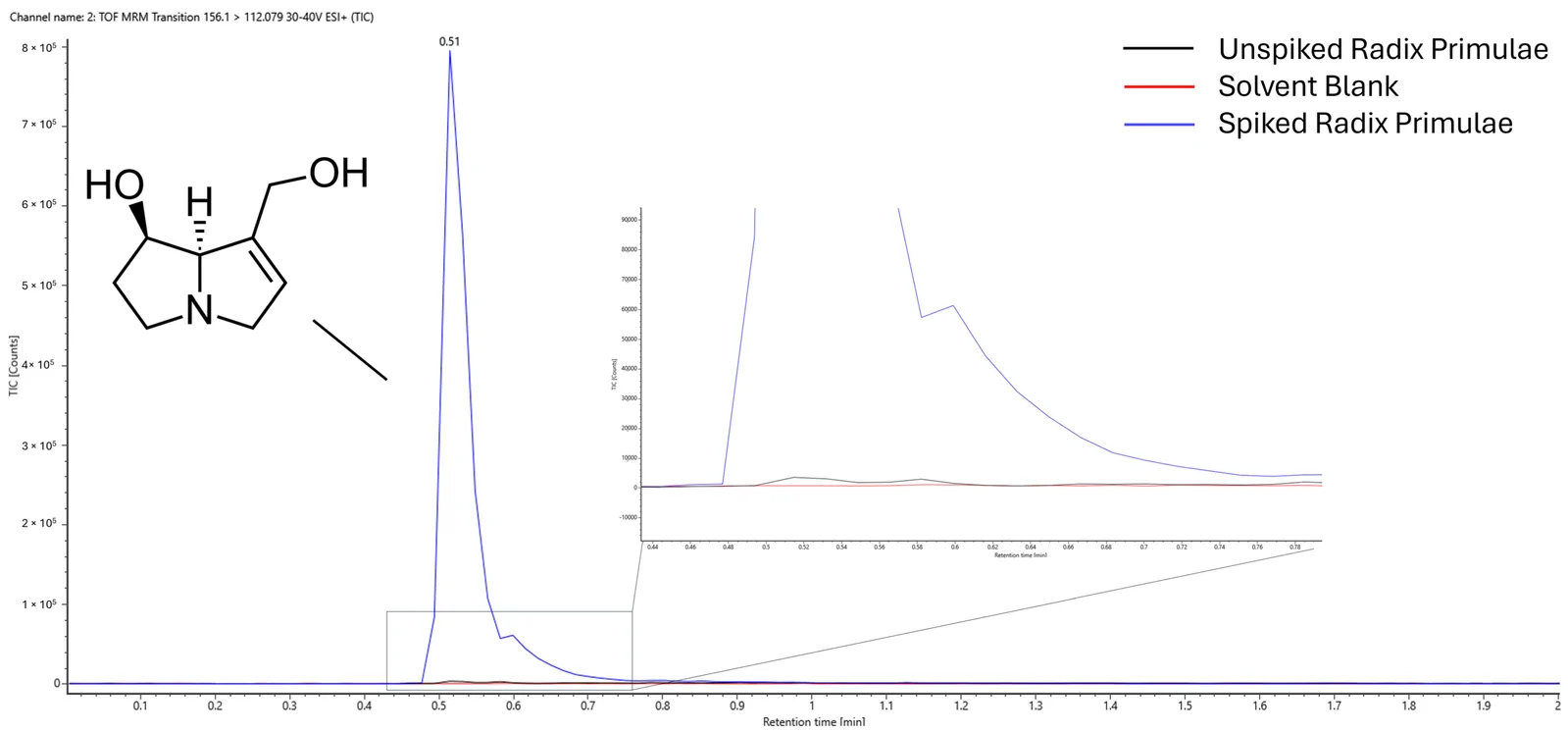
Overlay of chromatograms (measured using LC Method A (Section 3.2)) recorded for the retronecine transition *m*/*z* 156.1 → 112.079, showing a solvent blank (water/methanol; red), processed unspiked Radix Primulae (black), and Radix Primulae spiked before extraction at 250 µg/kg retronecine equivalents (blue). Both plant samples underwent the complete analytical procedure. The inset shows an enlarged view of the baseline around the retronecine retention time.

These recoveries reflect extraction, NZS enrichment, reduction/hydrolysis, work-up and LC–MS determination together. They provide evidence of overall process recovery for the investigated mixture but do not constitute an independently measured reaction yield or a mass balance for each PA. A representative chromatogram from the spike experiments is shown in Figure 2.

After 24 h, mean recoveries were 83.0%, 94.6% and 92.4% at 200, 250 and 300 µg/kg, respectively. The corresponding RSDs across the same three preparations per level were 2.86%, 2.03% and 2.07% (Table 1). Individual paired concentration changes relative to Day 1 ranged from −0.67% to +1.23% across all nine extracts. These results support consistent remeasurement over 24 h under the applied storage conditions (storage in the autosampler at 10 °C). Because the same prepared extracts were reanalysed, the comparison reflects extract storage and measurement variability together and does not independently establish inter-day precision of the complete extraction and conversion procedure.

### 2.2. Calibration Range and Limits of Detection and Quantification

Matrix-matched calibration was evaluated using six concentration levels (10, 100, 200, 300, 400 and 500 µg/L), each measured in triplicate on both days. Unweighted ordinary least-squares regression of all 18 peak-area measurements per day, with a freely fitted intercept, gave y = 7289.4216x + 111.7754 on Day 1 (R^2^ = 0.99936) and y = 7291.7512x − 4889.8978 on Day 2 (R^2^ = 0.99930).

LOD and LOQ were calculated using the ICH Q2(R2) σ/slope equations described in Section 3.8. The estimates were 14.94/45.27 µg/L on Day 1 and 15.58/47.21 µg/L on Day 2. The 10 µg/L calibration level was below the calculated LOD and LOQ and was therefore not considered a quantitative reporting level. All sample concentrations used for quantitative recovery assessment exceeded the corresponding daily LOQ.

Using the sample-specific combined dichloromethane volumes of 2.799–2.993 mL and 1.000 g plant portions, the corresponding plant-based LOD/LOQ estimates ranged from 41.82 to 44.72/126.72 to 135.50 µg/kg on Day 1 and 43.61 to 46.63/132.14 to 141.30 µg/kg on Day 2, without additional dilution. The spike-sample solutions ranged from 56.68 to 97.03 µg/L on Day 1 and from 56.32 to 96.80 µg/L after 24 h, exceeding the respective daily calculated LOQs.

### 2.3. Application to a Comfrey Sample

**Figure 3:**
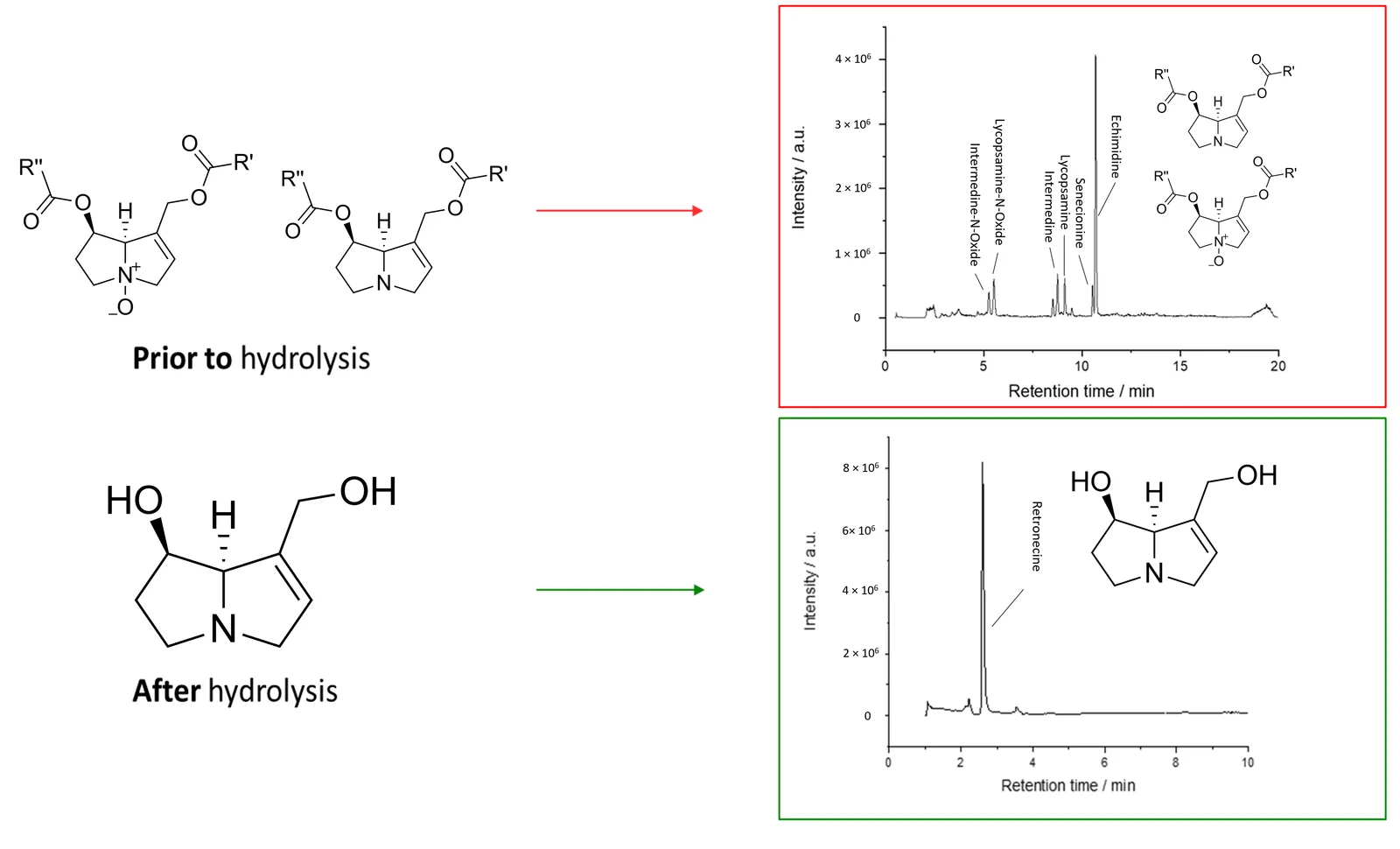
Comfrey analysed before and after reduction/hydrolysis using LC Method B (Section 3.2). Mobile phases: 0.1% formic acid in water and acetonitrile; flow rate: 0.45 mL/min.

A comfrey sample containing PAs was analysed by two approaches. For intact-PA determination, sample preparation followed Section 3.4 and Section 3.6 through reconstitution after NZS enrichment, without the subsequent reduction/hydrolysis. Echimidine, intermedine, intermedine N-oxide, lycopsamine, lycopsamine N-oxide and senecionine were identified and quantified by comparison with authentic standards measured under the same chromatographic conditions. Individual identities were assigned using retention behaviour and mass-spectrometric signals (supporting identification data for the six intact PAs/PANOs are provided in Table S1). The same sample was analysed using the complete conversion workflow. Both approaches yielded 4.2 mg/kg retronecine equivalents without spiking. For comparison, the individually quantified intact PAs were converted to retronecine equivalents on a molar basis and summed. The comparison in Figure 3 supports agreement for this particular sample and detected PA profile. The chromatograms in Figure 2 and Figure 3 were acquired using different LC methods (Methods A and B, Section 3.2), so the different necine-base retention times are not directly comparable.

### 2.4. Product Identity and Analytical Selectivity

The method is class-specific rather than compound-specific. NZS enrichment provides cleanup before conversion, while retention-time agreement, accurate precursor mass and diagnostic product ions are used to assess the identity of the final marker against authentic retronecine. Heliotrine and heliotrine N-oxide are expected to yield heliotridine rather than retronecine. Retronecine and heliotridine are stereoisomeric necine bases with the same elemental composition, so accurate precursor mass alone cannot distinguish them. No separate heliotridine response factor or chromatographic resolution from retronecine is established by the present data. Reporting the combined response as retronecine equivalents is therefore an operational quantification convention. The mixture recovery cannot establish the individual fate or conversion efficiency of heliotrine or heliotrine N-oxide.

The validation mixture included monoesters (intermedine, lycopsamine and heliotrine), an open-chain diester (echimidine) as well as a macrocyclic diester (senecionine), together with their N-oxides. This provides structural diversity within the tested mixture but does not establish identical enrichment or conversion for all congeners. Otonecine-type and saturated necine-base PAs were not validated by this workflow and are outside its demonstrated scope.

### 2.5. Matrix Effects and Scope of Validation

Matrix-matched external calibration was used without an internal standard. Matching the final calibration and sample matrix can compensate for systematic ion suppression or enhancement associated with that matrix. It does not correct every sample-specific change or independently establish the magnitude of matrix effects. Recovery was assessed using PAs/PANOs added before extraction and therefore reflects the complete procedure.

Formal validation was performed in *Radix Primulae*. The comfrey comparison is an additional application in a chemically different matrix, not a full second-matrix validation. In particular, calibration in *Radix Primulae* cannot be assumed to compensate identically for the comfrey matrix.

### 2.6. Comparison with Published Methods in Plant Matrices

The use of a common marker for PA quantification has already been explored in plant materials through several analytical strategies. Zhang et al. [28] converted retronecine ester-type PAs into a common coloured derivative using o-chloranil and Ehrlich’s reagent and applied the HPLC method to Senecio scandens. They reported solution LOD and LOQ values of 0.26 and 0.79 nmol/mL, respectively. Xiong et al. [29] subsequently described an oxidative derivatization method for herbs using HPLC–DAD at 223 nm, with recoveries of 94.6–100.7% and intra- and inter-day variations below 4.6%. Their derivatization required 3 h of heating, whereas the conversion step used here was performed for 30 min at 4 °C. This comparison concerns the reaction step only, because the present workflow also includes NZS enrichment and solvent work-up. These earlier studies established the feasibility of marker-based quantification with optical detection. The present method instead couples enrichment to necine-base conversion and LC-MS detection.

A particularly relevant comparison is the study of Tsiokanos et al. [24], which combined UHPLC–HRMS profiling with retronecine/heliotridine-based quantification directly in crude medicinal-plant extracts. Their investigation included *Alkanna* species and PA-containing plants from the Fabaceae and Compositae families and assigned 105 PAs across the investigated extracts. In four *Alkanna* species, the compounds detected were outside the 35-PA target list considered in that study, illustrating how a restricted targeted panel can miss part of a botanical PA profile. Their approach also combined necine-base results with profiling-derived correction factors to estimate PA mass content. The present workflow shares the aim of reducing dependence on individual PA standards but places selective NZS enrichment before conversion. Its distinct contribution is therefore the evaluation of this enrichment-conversion sequence, rather than the first use of retronecine-based quantification in plant extracts.

The enrichment component builds on our previous NZS study in verbena extracts [14], which reported recoveries of 72–95% for intact PAs and 30–70% for PANOs and solution LOQs of 0.64–4.5 ng/mL. In that study, enriched compounds were quantified individually; here, enrichment is followed by conversion to a necine-base response. The present Day 1 mean mixture recoveries of 83.0–94.2% show that the combined procedure can retain a substantial proportion of the spiked PA-derived response, but they do not establish identical recovery of each congener or isolate the contribution of the sorbent. Activation, washing and elution conditions were adopted from the established procedure without a new systematic study of sorbent amount, pH, ionic strength, contact time, or competing constituents.

Targeted methods provide a complementary perspective because they retain compound-specific information. Kaltner et al. [30] developed LC–ESI–MS/MS determination of 44 PAs/PANOs in teas and herbal teas, with LODs of 0.1–7.0 µg/kg, precision values of 0.7–16.1% and recoveries of 60.7–128.8% for 40 analytes. Their method development included extraction and SPE optimisation and chromatographic separation of isomers. Such methods can relate contamination to a particular PA profile, but require an extensive set of individual standards and compound-specific calibration. The present method uses retronecine as the routine calibrant after conversion, while retaining the need for individual standards during validation and reference measurements. Its mixture recovery is therefore not directly interchangeable with the congener-specific recovery ranges reported for targeted methods.

Taken together, these plant-based studies position the present workflow as an integration of selective cleanup with an established sum-parameter concept. Its analytical value lies in concentrating the PA/PANO fraction before conversion and reducing the number of standards needed for routine marker quantification. The agreement between the conversion-based and intact-PA results in comfrey supports this combined strategy for the investigated sample. Conversion nevertheless removes the individual PA profile and results expressed as retronecine equivalents remain distinct from the mass sum of intact regulated PAs. The method is therefore most appropriately used alongside targeted analysis when both an aggregate response and compound-specific confirmation are required.

## 3. Materials and Methods

### 3.1. Reagents and Plant Materials

Acetonitrile (ACN), methanol, formic acid (FA) and water, all of LC–MS grade, and PTFE syringe filters (0.2 µm pore size, 13 mm diameter) were purchased from Fisher Scientific International Inc. (Pittsburgh, PA, USA).

Nano zirconium silicate (NZS; batch MKBC0475) and sulfuric acid (ACS reagent, 95.0–98.0%) were purchased from Sigma-Aldrich (St. Louis, MO, USA). Sodium hydroxide solution (5 M) was purchased from Carl Roth GmbH + Co. KG (Karlsruhe, Germany).

*Radix Primulae* was obtained from Kräuter Schulte GmbH (Gernsbach, Germany). The material was supplied under lot number 330029322. *Radix Symphyti* was supplied by Kottas Pharma (Vienna, Austria) under lot number W24201890.

PA standards, namely lycopsamine, lycopsamine N-oxide, heliotrine, heliotrine N-oxide, intermedine, intermedine N-oxide, echimidine, echimidine N-oxide, senecionine and senecionine N-oxide were purchased from PhytoLab GmbH & Co. KG (Vestenbergsgreuth, Germany).

Retronecine was purchased from Bartelt GmbH (Graz, Austria).

### 3.2. Instrumentation

LC–HRMS analysis was performed using an ACQUITY Premier system coupled to a Xevo G3 quadrupole time-of-flight mass spectrometer (Waters, Milford, MA, USA). Two LC methods were used, as specified below. Mass-spectrometric detection used positive electrospray ionisation. The transition *m*/*z* 156.1012 → 112.079 was used for quantification of retronecine, while *m*/*z* 156.1012 → 138.091 served as the qualifier transition. Reported source settings were a capillary voltage of 3.0 kV, a cone voltage of 40 V, a source temperature of 100 °C, a desolvation temperature of 250 °C, a cone gas flow of 100 L/h and a desolvation gas flow of 1000 L/h. Collision energy was 30–40 V. Nitrogen was used as the collision gas. Sodium formate was used before analysis to check mass calibration, with lock-mass (Leucine-Enkephaline) correction during acquisition.

LC Method A was used for all experiments except the analyses of the comfrey sample, which were performed using LC Method B. Separation used a ZORBAX RRHD C18 column (2.1 × 100 mm, 1.8 µm; Agilent Technologies, Santa Clara, CA, USA), 0.1% formic acid in water (A) and acetonitrile (B), at 0.45 mL/min. The gradient (min/% B) was 0/1, 2/25, 4/35, 6/35, 10/95, 13/95, 13.1/1 and 17/1. The reported injection volume was 2 µL, column temperature 50 °C and autosampler temperature 10 °C.

LC Method B was used for Figure 3. Separation used a ZORBAX RRHD C18 column (2.1 × 100 mm, 1.8 µm; Agilent Technologies, Santa Clara, CA, USA), 0.1% formic acid in water (A) and acetonitrile (B), at 0.45 mL/min. The gradient (min/% B) was 0/1, 5/1, 10/35, 15/95, 17/95, 17.1/1 and 20/1. The reported injection volume was 2 µL, column temperature 50 °C and autosampler temperature 10 °C.

### 3.3. Preparation of Spiked Plant Material

After grinding the plant material using a Retsch ZM200 mill (Retsch GmbH, Haan, Germany), the sample was homogenised by passing it through a sieve with a pore size of 1 mm. Portions of 1.000 g were spiked before extraction at 200, 250 and 300 µg/kg retronecine equivalents. The PA/PANO mixture comprised lycopsamine, lycopsamine N-oxide, heliotrine, heliotrine N-oxide, intermedine, intermedine N-oxide, echimidine, echimidine N-oxide, senecionine and senecionine N-oxide. Three independent preparations were made at each level. Each final extract was analysed by duplicate injections on Day 1 and again after 24 h.

The spike concentrations were expressed as retronecine equivalents using the molecular masses of the individual PA/PANO standards.

The nominal total amounts added to each 1.000 g plant portion were 0.200, 0.250 and 0.300 µg retronecine equivalents at the respective spike levels. Sample-specific combined dichloromethane volumes were used when calculating plant concentrations (Section 3.8).

### 3.4. Sample Extraction

The spiked sample was extracted with 5 mL of 0.1 M sulfuric acid by ultrasound-assisted extraction for 15 min. Preliminary experiments confirmed that a single extraction step was sufficient, as performing two or three consecutive extraction cycles did not result in major changes in the extracted content. The extract was then centrifuged at 3500 rpm for 15 min. Subsequently, 500 µL of the resulting supernatants was mixed with 500 µL of 0.1 M sodium hydroxide. The resulting solution was then subjected to enrichment using the dispersive solid-phase extraction procedure described in Section 3.6.

### 3.5. Calibration

Matrix-matched calibration used retronecine added to a processed extract of the same unspiked *Radix Primulae* material used for validation. Before preparing the calibrators, the unspiked matrix was analysed and showed no detectable retronecine/PA-derived signal at the relevant retention time; no background subtraction was therefore applied to this calibration batch. The unspiked matrix was assessed separately and was not included as a zero level in the regression, which was not forced through the origin. The calibration levels were 10, 100, 200, 300, 400 and 500 µg/, with three injections at each level on both measurement days. The 10 µg/L level was retained as a low-concentration calibration point in the regression but was not considered the lower quantitative reporting limit based on the subsequently calculated LOD and LOQ. A separate unweighted ordinary least-squares regression with an intercept was fitted to all 18 responses from each day. Day 1 samples were quantified using the Day 1 regression and the 24 h remeasurements using the Day 2 regression.

### 3.6. SPE Method

The NZS enrichment conditions were adopted from the previously established procedure [14]. Before use, 500 mg of NZS was activated by adding 500 µL of a 1:1 methanol/water solution, followed by vortexing for 1 min and centrifugation at 4000× *g*. After discarding the supernatant, 500 µL of the plant extract/sodium hydroxide mixture from Section 3.4 was added directly onto the activated NZS. The suspension was again vortexed for 1 min and centrifuged at 4000× *g* and the supernatant was discarded. The sorbent was then washed once with 500 µL of pure methanol using the same vortexing and centrifugation parameters. Elution of the retained analytes was performed using 30% formic acid in methanol. For optimal recovery, the elution was divided into three steps, each using 500 µL of eluent. After each addition, the mixture was vortexed for 1 min and centrifuged at 4000× *g* and the eluates were collected. The three fractions (total volume 1.5 mL) were pooled, evaporated to dryness by vacuum centrifugation and reconstituted in 50 µL of a 1:1 methanol/water mixture. The sample was then subjected to the reduction and hydrolysis procedure using LiAlH_4_, as described in Section 3.7.

### 3.7. Reduction and Hydrolysis Process

**Figure 4:**
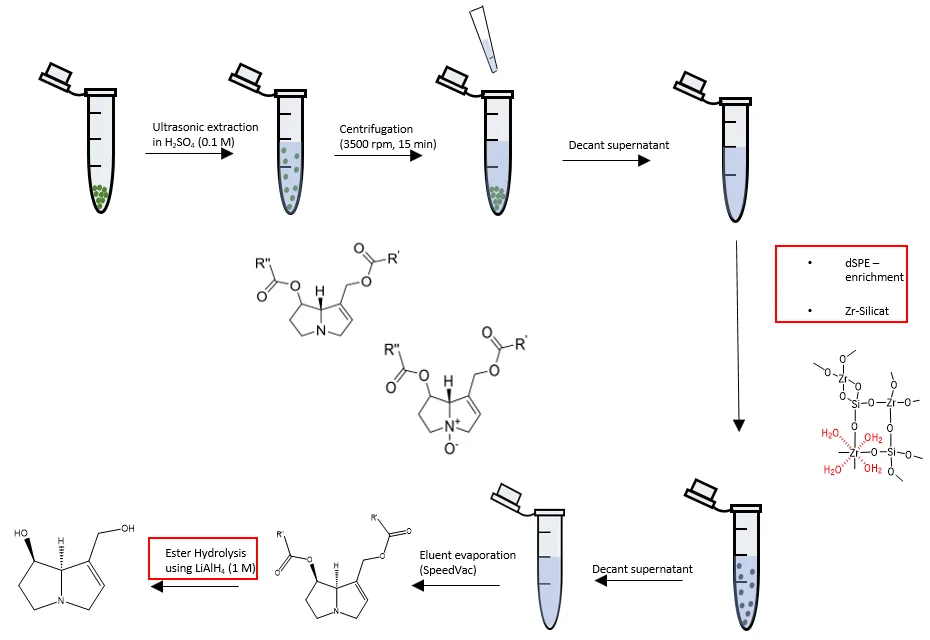
Analytical workflow comprising NZS-based dispersive SPE enrichment followed by one-pot LiAlH_4_ reduction/hydrolysis and LC–HRMS determination of the necine-base response as retronecine equivalents.

For the one-pot reduction and hydrolysis, 0.6 mL of LiAlH_4_ in THF (1 M) was added to the dried and reconstituted sample. The mixture was briefly vortexed and subsequently cooled for 30 min at 4 °C. After the cooling step, 0.2 mL of 10% (*w*/*v*) NaOH, 0.5 g of Na_2_SO_4_ and 1 mL of CH_2_Cl_2_ were added to the reaction mixture (workflow adapted from [28]). The mixture was vortexed again and the organic phase (CH_2_Cl_2_) was collected. Two additional extractions were performed using 1 mL of CH_2_Cl_2_ each. The volume of the combined organic phases was determined gravimetrically by transferring the extracts into a sealable vial and weighing them. The total volume was then calculated based on the density of dichloromethane. Then, 1 mL of the combined organic extract was evaporated to dryness under a gentle stream of nitrogen. The residue was resuspended in 50 µL of a 1:1 methanol/water solution and subsequently analysed by LC-MS. The same final extracts were remeasured after 24 h. No separate dilution-integrity study was performed. Figure 4 provides a graphical overview of the complete enrichment and reduction workflow.

### 3.8. Method Validation

Calibration was assessed using six concentration levels between 10 and 500 µg/L and three injections per level on each of two measurement days. The regression slope, intercept, R^2^, residual standard deviation, residuals and back-calculated concentrations were evaluated. Calibration fit was evaluated separately from recovery and between-preparation precision.

Three independent preparations were analysed at each level, with two injections per preparation on Day 1 and two further injections of the same extract after 24 h. Mean recovery, observed range and between-preparation RSD were calculated from the three preparation results per level and day. RSD was calculated as 100 × s/mean using the sample standard deviation s (n − 1 denominator). Paired changes after 24 h were calculated as 100 × (C24h − CDay1)/CDay1 for each extract. Results are presented in Table 1 and Section 2.1.

In line with the recommended statistical approach, the LOD was determined as LOD = 3.3 (σ/S) and the LOQ as LOQ = 10 (σ/S), where σ represents the residual standard deviation of the calibration regression and S the slope of the calibration curve. These calculations were performed using the regression data obtained from the six-point calibration. The resulting LOD and LOQ values are expressed in µg/L and therefore reflect the method’s inherent analytical sensitivity based on the ICH-recommended statistical model.

The unspiked matrix was assessed separately and was not entered as a zero calibrator. Plant-based estimates used the same aliquot, volume and sample-mass factors as the spike calculations.

Selectivity was assessed by comparison of a solvent blank, unspiked matrix, spiked matrix and authentic samples with the reference standard.

The PA-derived necine-base response was expressed as retronecine equivalents in the dried plant material using the following calculation. Any additional sample dilution factor was applied to the measured extract concentration before calculating the plant concentration:

$$\left(\frac{\frac{c_{l}}{20000}\cdot V_{CH_{2}{Cl}_{2}}\cdot 20}{m_{s}}\right)\cdot 10^{6}=c_{s}$$

$c_{l}$—Necine-base concentration in the measured extract, expressed as retronecine equivalents/µg/L;
$V_{CH_{2}{Cl}_{2}}$—Measured volume of the combined dichloromethane extract/mL;
$m_{s}$—Mass of the plant material before extraction/mg;
$c_{s}$—PA-derived necine-base concentration in plant material, expressed as retronecine equivalents/µg/kg.

## 4. Conclusions

The combined NZS enrichment and LiAlH_4_ reduction/hydrolysis workflow provides a sum-parameter approach for the investigated retronecine- and heliotridine-type PAs/PANOs, with results expressed as retronecine equivalents. Three independent *Radix Primulae* preparations at each of 200, 250 and 300 µg/kg, measured in duplicate, gave Day 1 mean recoveries of 83.0%, 94.2% and 92.5% and between-preparation RSDs of 3.12%, 1.55% and 1.96%. Calculated LOD/LOQ estimates were 14.94/45.27 µg/L on Day 1 and 15.58/47.21 µg/L on Day 2; all spike-sample solutions exceeded the respective daily calculated LOQs. Remeasurement of the same extracts after 24 h gave paired concentration changes of −0.67% to +1.23%. These results support recovery and short-term remeasurement consistency in the investigated matrix.

The contribution of this work is the integration and evaluation of established enrichment and conversion procedures in a botanical matrix. Agreement with the intact-PA reference approach in comfrey supports an additional real-sample application. Formal quantitative validation remains limited to *Radix Primulae* and transfer to other matrices requires additional evaluation. The workflow reduces dependence on individual PA standards for routine sum quantification, while sacrificing compound-specific information.

## Figures and Tables

**Table 1:** Recovery and precision for three independent preparations per spike level, measured on Day 1 and remeasured after 24 h (RE—retronecine equivalents, *n*—number of independent sample preparations).

Spike (µg/kg RE)	*n*	Day 1 Recovery (%)	Day 1 RSD (%)	24 h Recovery (%)	24 h RSD (%)
200	3	83.0 (80.6–85.8)	3.12	83.0 (81.1–85.6)	2.86
250	3	94.2 (93.3–95.9)	1.55	94.6 (92.7–96.5)	2.03
300	3	92.5 (90.4–93.7)	1.96	92.4 (90.2–93.8)	2.07

## Data Availability

The data presented in this study are available from the corresponding author upon reasonable request.

## Supplementary Materials

The following supporting information can be downloaded at: https://www.mdpi.com/article/10.3390/molecules31203590/s1, Table S1: Identification data for the six PAs/PANOs assigned in the comfrey extract before reduction/hydrolysis (Figure 3; LC Method B).
