# Supplementary material for: Simplifying PA Quantification: A Unified Enrichment and Reduction Workflow for Retronecine-Based Quantification of Pyrrolizidine Alkaloids in Phyto Samples
Source: Molecules. 2026 Oct 9;31(20):3590. doi: 10.3390/molecules31203590 (PMC13707566; doi:10.3390/molecules31203590)
Supplement: Supplementary file 1 [file molecules-31-03590-s001.zip › molecules-4604842-supplementary.pdf]

Supplementary identification data for intact pyrrolizidine alkaloids

Table S1. Identification data for the six PAs/PANOs assigned in the comfrey extract before reduction/hydrolysis (Figure 3; LC Method B).

Compound	Standard RT (min)	Sample RT (min)	Measured $[M+H]^+$ m/z	Observed product ions m/z
Intermedine N-oxide	5.3	5.3	316.1748	111, 138
Lycopsamine N-oxide	5.5	5.5	316.1751	111, 138
Intermedine	8.8	8.8	300.1807	156
Lycopsamine	9.1	9.1	300.1806	138, 156
Senecionine	10.5	10.5	336.1804	120, 138
Echimidine	10.7	10.7	398.2169	120, 220

Acquisition mode: MS^E (50 – 500 m/z) Collision ramp: 30 – 40 V.
